# Microglial VPS35 deficiency regulates microglial polarization and decreases ischemic stroke-induced damage in the cortex

**DOI:** 10.1186/s12974-019-1633-y

**Published:** 2019-11-26

**Authors:** Shi-Yang Ye, Joanna E. Apple, Xiao Ren, Fu-Lei Tang, Ling-Ling Yao, Yong-Gang Wang, Lin Mei, Yuan-Guo Zhou, Wen-Cheng Xiong

**Affiliations:** 1 Center of Molecular Biology, State Key Laboratory of Trauma, Burn and Combined Injury, Research Institute of Surgery, Daping Hospital, Third Military Medical University, Chongqing, China; 2 Department of Neurosciences, School of Medicine, Case Western Reserve University, Cleveland, OH USA; 3 Department of Neuroscience and Regenerative Medicine, Medical College of Georgia, Augusta University, Augusta, GA USA; 4 Department of Neurology, Renji Hospital, Shanghai Jiaotong University, Shanghai, China

**Keywords:** VPS35, Microglia, Ischemic brain injury, CX3CR1

## Abstract

**Background:**

Vacuolar sorting protein 35 (VPS35), a critical component of retromer, is essential for selective endosome-to-Golgi retrieval of membrane proteins. It is highly expressed in microglial cells, in addition to neurons. We have previously demonstrated microglial VPS35’s functions in preventing hippocampal, but not cortical, microglial activation, and in promoting adult hippocampal neurogenesis. However, microglial VPS35’s role in the cortex in response to ischemic stroke remains largely unclear.

**Methods:**

We used mice with VPS35 cKO (conditional knockout) in microglial cells and examined and compared their responses to ischemic stroke with control mice. The brain damage, cell death, changes in glial cells and gene expression, and sensorimotor deficits were assessed by a combination of immunohistochemical and immunofluorescence staining, RT-PCR, Western blot, and neurological functional behavior tests.

**Results:**

We found that microglial VPS35 loss results in an increase of anti-inflammatory microglia in mouse cortex after ischemic stroke. The ischemic stroke-induced brain injury phenotypes, including brain damage, neuronal death, and sensorimotor deficits, were all attenuated by microglial VPS35-deficiency. Further analysis of protein expression changes revealed a reduction in CX3CR1 (CX3C chemokine receptor 1) in microglial VPS35-deficient cortex after ischemic stroke, implicating CX3CR1 as a potential cargo of VPS35 in this event.

**Conclusion:**

Together, these results reveal an unrecognized function of microglial VPS35 in enhancing ischemic brain injury-induced inflammatory microglia, but suppressing the injury-induced anti-inflammatory microglia. Consequently, microglial VPS35 cKO mice exhibit attenuation of ischemic brain injury response.

## Background

Ischemic stroke is one of the most common brain diseases, accounting for 70% of cerebrovascular diseases [1]. It is the fourth leading cause of death in the USA and Europe, and a major cause of adult disability [2]. It is thus of considerable interest to investigate the cellular and molecular mechanisms of ischemic brain damage. Many progresses have been achieved, but, it remains to be a detrimental disease without an effective treatment in clinic. Thus, it is necessary to further investigate its pathological mechanisms for identification of novel intervention targets for the development of a better therapy.

Ischemic brain injury triggers a complex pathophysiological cascade, which includes impaired blood flow, hypoxia, oxidative stress, glutamate excite-toxicity, and inflammation [1]. During an ischemic event, impaired blood flow in the brain occurs first, which results in a reduced delivery of oxygen and glucose, leading to the energy depletion and neuronal death that is largely located in the ischemic core [3, 4]. There are glial responses to the neuronal death or damaged neurons mainly in the peri-infarct region, which include reactive astrogliosis and microglial activation.

Microglia, the resident immune cells of the brain, react quickly to ischemic brain injury with transcriptional regulation and morphological changes [5]. Based on their transcriptional changes, microglia have been classified as M1, M2, and recently DAM (damage-associated microglia) [6, 7]. The different types of microglia appear to have different functions during ischemic brain injury, whereas M2 microglial cells show protective effects during ischemic injury, M1 microglia appear to play a detrimental role in this event [8, 9]. The functions of DAM in response to the ischemic injury or its relationship with M1 microglia remain to be investigated.

VPS35 (Vacuolar sorting protein 35), a critical component of retromer, is essential for selective endosome-to-Golgi retrieval of transmembrane proteins [10]. VPS35 is a ubiquitous protein with different levels of expression in different cells of the central nervous system, including microglia. In the central nervous system, VPS35 has been shown to be involved in many key cellular physiological processes. For example, AMPA receptor mediated neurotransmission [11], mitochondrial fusion/fission dynamics [12], and β-amyloid (Aβ) metabolism [13]. Pathologically, VPS35-deficiency is believed to increase the risk of neurodegenerative diseases, including Alzheimer’s disease (AD) and Parkinson’s disease (PD) [14]. Mutations in VPS35 gene have been identified in patients with autosomal dominant PD [15–17] or early onset AD [18]. VPS35 loss in mouse models causes PD-like deficits as well as enhances AD-like neuropathology in Tg2576, an AD mouse models [13, 14, 19]. Interestingly, the microglial VPS35 is decreased in the brain of AD patients [20]. We have previously shown that microglial VPS35 knocking out in mouse model results in a selective microglial activation in the hippocampus and interferes with the neurogenesis in adult dentate gyrus [21]. However, the function of microglial VPS35 in the cortex in response to ischemic stroke remains elusive.

Here, we report that microglial VPS35 loss regulates microglial polarization in the cortical brain after ischemic stroke. Ischemic stroke-induced injury is diminished in microglial VPS35 conditional knockout (cKO) mice. In addition, ischemic stroke-induced increase of CX3CR1 receptor levels is abolished in microglial VPS35-deficient mice, implicating CX3CR1 as a potential cargo of VPS35. Taken together, these results suggest an unrecognized function of microglial VPS35 in suppressing microglial polarization change (from pro-inflammatory to anti-inflammatory), in line with the view for pro-inflammatory microglia to play a “neurotoxic” role in ischemic brain injury.

## Methods

### Animals

CX3CR1^Cre-ER^ mice in C57BL/6 background were purchased from the Jackson Laboratory (stock number: 00790). VPS35^flox/flox^:CX3CR1^Cre-ER/+^ mice (termed as VPS35^CX3CR1^ in this study) were generated as described previously [21]. Mice were housed under constant 12-h light-dark cycle and fed a diet of standard rodent chow. All animal experimental procedures were approved by the Animal Subjects Committees at Case Western Reserve University and Augusta University according to US National Institutes of Health guidelines.

### Photothrombotic ischemic stroke

Ischemic damage of sensorimotor cortex in mice was induced by photothrombotic ischemia as described previously [22]. Briefly, mice (control and mutant) were anesthetized by ketamine (100 mg/kg) and intraperitoneally injected with same amount of Rose Bengal (10 mg/ml and 10 μl/g body weight). Mice with an exposed skull were then placed under the stereotaxic instrument with their heads firmly secured. Ten minutes after Rose Bengal injection, a cold light was turned on specifically on the sensorimotor cortex region for 15-min photo illumination.

We chose 10 min after Rose Bengal injection for photo illumination, because its concentration in the blood (measured by Rose Bengal-BSA binding assay) reached a peak level [23]. To make sure same size of area exposed to the light and to prevent photo illumination of the animal by any other source of light, an opaque patch with a hole (2-mm diameter) in the center was put under the skin of the skull, and the end of illuminator fiber (with 150-W intensity) was tightly attached to the center of the patch.

### Immunofluorescence staining

Immunofluorescence staining analysis was performed on 40 μm free-floating sections. Primary antibodies, including chicken anti-GFP (Aves Labs, GFP-1020), goat anti-CD206 (R&D Systems, AF2535), rabbit anti-Cleaved Caspase-3 (Cell Signaling, 9661), mouse anti-Caspase-3 (Novus Biologicals, 31A1067), mouse anti-NeuN (Millipore, MAB377), rabbit anti-Tmem119 (Abcam, ab209064), mouse anti-LPL (Abcam, ab21356), rabbit anti-CX3CR1(Abcam, ab8021), rabbit anti-GFAP (Abcam, ab7260), mouse anti-iNOS (inducible nitric oxide synthase) (Abcam, ab49999), and goat anti-Iba1 (Abcam, ab5076), were used. Corresponding secondary antibodies, including 488- and 594-conjugated secondary antibodies, were purchased from Thermo Fisher Scientific, Alexa Fluor conjugates.

All images were processed with Image J for quantification analysis. The means were calculated from 3 randomly selected microscopic fields in the ipsilateral and contralateral cortex of each section, respectively, and 3 consecutive sections were analyzed for each brain. Data are expressed as mean numbers of cells per square millimeter.

### Real-time PCR

Total RNAs were isolated from ischemic brains using the RNAeasy Mini Kit (Qiagen) according to the manufacturer’s instructions. Five micrograms was used to synthesize the first strand of cDNA using the Superscript First-Strand Synthesis System for RT-PCR (Invitrogen). PCR was performed on the Opticon 2 Real-Time PCR Detection System (Bio-Rad) using corresponding primers (Table 1) and SYBR gene PCR Master Mix (Invitrogen). The cycle time values were normalized to GAPDH of the same sample. The expression levels of the mRNAs were then reported as fold changes over control.

**Table 1:** Primers for real-time polymerase chain reaction

Gene	Primer
CD86	Forward: GACCGTTGTGTGTGTTCTGG Reverse: GATGAGCAGCATCACAAGGA
CD206	Forward:CAAGGAAGGTTGGCATTTGT Reverse: CCTTTCAGTCCTTTGCAAGC
iNOS	Forward: CAAGCACCTTGGAAGAGGAG Reverse: AAGGCCAAACACAGCATACC
CD32	Forward: AATCCTGCCGTTCCTACTGATC Reverse: GTGTCACCGTGTCTTCCTTGAG
Arg1	Forward: TCACCTGAGCTTTGATGTCG Reverse: CTGAAAGGAGCCCTGTCTTG
Ym1/2	Forward: CAGGGTAATGAGTGGGTTGG Reverse: CACGGCACCTCCTAAATTGT
IL-6	Forward: CTCTGCAAGAGACTTCCATCCA Reverse: GACAGGTCTGTTGGGAGTGG
IL-1β	Forward: CTGATGCAGGTCCCTATGGT Reverse: GCAGGATTTTGAGGTCCAGA
IL-10	Forward: CCAAGCCTTATCGGAAATGA Reverse: TCCTCACAGGGGAGAAATCG
IL-4	Forward: ATGATGCAGGTCCCTATGGT Reverse: GCAGGATTTTGAGGTCCAGA
TGF-β	Forward: TGCGCTTGCAGAGATTAAAA Reverse: CGTCAAAAGACAGCCACTCA
TNF-α	Forward: CAGGCGGTGCCTATGTCTC Reverse: CGATCACCCCGAAGTTCAGTAG

### Neurological functional tests

The two groups of mice (cKO and control) were trained for 1 week before the test. All the tests were measured before, 6 h, 1, 3, and 7 days after ischemia/stroke. Modified neurological severity score assessment, adhesive removal test, and foot fault test were performed as described previously [24]. Neurological function assessments were performed by investigators who were blinded to the experimental groups. Neurological function was graded on a scale of 0 to 14 (normal = 0; maximal deficit = 14). The higher the score, the more serious the neurological impairment.

### TTC staining and Nissl staining to assess infarct volume

The infarct volume was accessed by TTC and Nissl staining as described previously [24]. Briefly, after anesthesia, mouse was euthanized, and its whole brain was removed. For TTC staining, the brain slice sections (750-μm thick, coronal) were incubated with 2% TTC (2,3,5-triphenyltetrazolium chloride) in PBS solution at 37 °C for 10 min, then fixed with 4% paraformaldehyde for 30 min, and washed with PBS for 5 min for 3 times. For Nissl staining, the brain sections (40-μm think, coronal) were incubated with 1% toluidine blue in PBS at 60 °C for 40 min, and then washed, dehydrated, and sealed for imaging analysis as described previously [25].

The infarct volume in TTC-stained or Nissl-stained sections was measured by a blinded observer using National Institutes of Health Image J software. The infarct area and volume on each slice were calculated based on the following equations according to a previous publication [26]: Infarct area = Contralateral hemisphere area − healthy area of ipsilateral hemisphere. Infarct volume = Infarct area × thickness of slice.

### Statistical analysis

All data were expressed as means ± SEM. The data were statistically analyzed by two-way analysis of variance (ANOVA), followed by Tukey’s test for pairwise comparisons by using Graph Pad Prism v7.0 and Sigma Plot 13.0 software. In the case of significance, a further ad hoc two-tailed Student’s *t* test was applied. *P* value < 0.05 was considered statistically significant.

## Results

### Decreases in infarct volume, neuronal death, and reactive astrogliosis in microglial VPS35-cKO mice in response to cortical ischemia/stroke

**Fig. 1:**
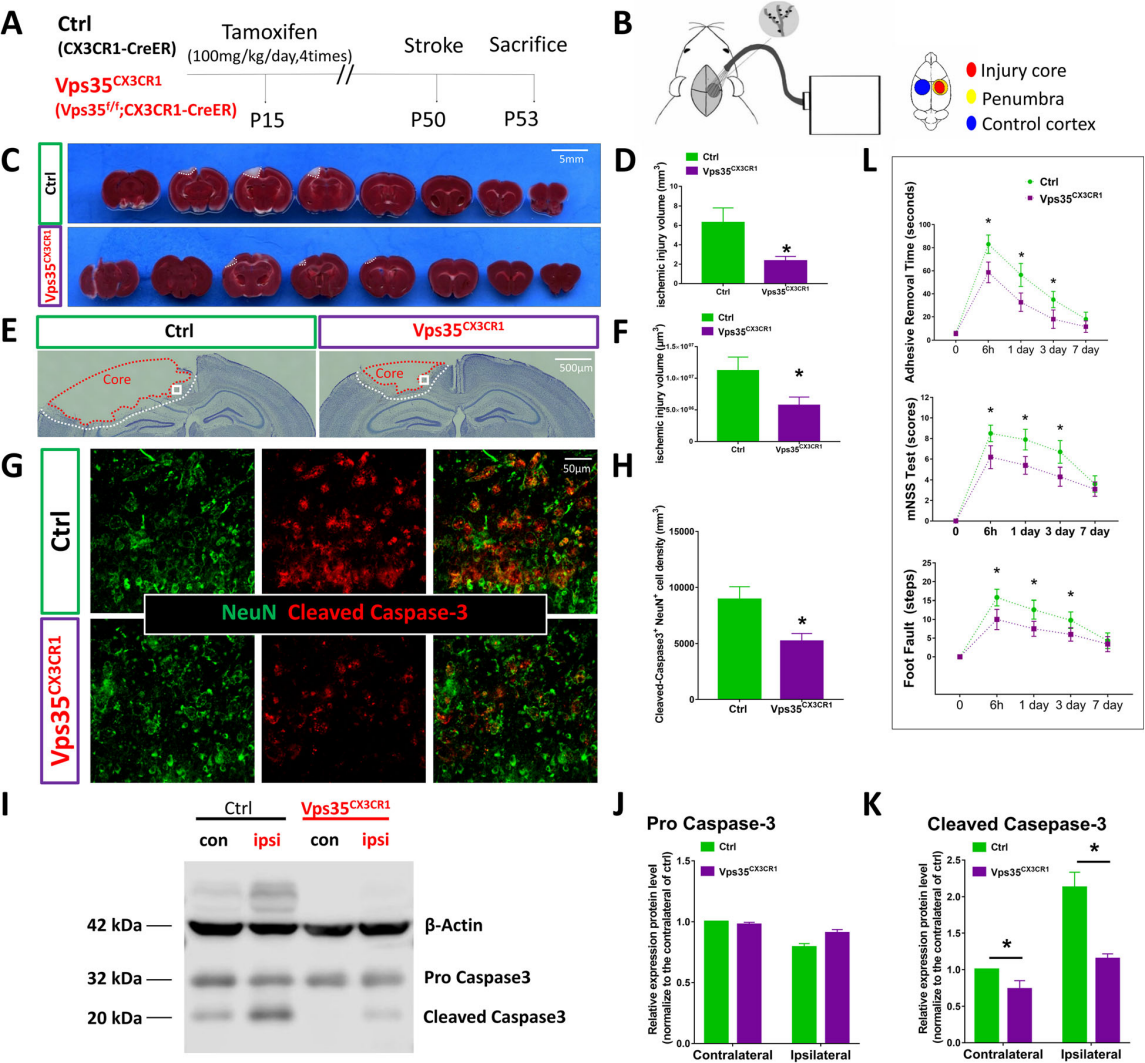
Decreases of infarct volume and neuronal death in microglial VPS35 CKO cortex in response to ischemia/stroke. **a** Schematic of the time course for stamoxifen injection and ischemic stroke injury in microglia-specific VPS35 conditional knockout (CKO) mice (VPS35^CX3CR1^). Tamoxifen (100 mg/kg) was injected (i.p.) into VPS35^CX3CR1^ and control (CX3CR1-CreER) mice at P15. Photothrombotic stroke was induced at P50, and mice were sacrificed 3 days after stroke. Their brain sections were subjected to TTC (2,3,5-triphenyltetrazolium chloride) staining (**c**, **d**), Nissl staining (**e**, **f**), or co-immunostaining analyses (**g**, **h**). The homogenates of the cortex were subjected to Western blot analysis (i–k). **b** Illustration of the photothrombotic stroke method. The mouse was injected (i.p.) with Rose Bengal (0.1 mg/g body weight), and 10 min later, its skull at the region of somatosensory cortex was exposed to a cold light to induce ischemic infarct. The ischemic core, peri-infarct, and contralateral cortex are indicated. **c** Representative images of TTC staining analysis. Bar, 5 mm. d Quantitative analysis of ischemic injury volumes. **e**, **f** Representative images of Nissl staining and quantitative analyses of injured brain regions. Bar, 500 μm. **g**, **h** Representative images of co-immunostaining analysis with antibodies against cleaved caspase 3 (a cell death marker) and NeuN (a neuronal marker) (**g**). Bar, 50 μm. Quantitative analysis (mean ± SEM; *n* = 5/group; *P < 0.05) was included (**h**). **i**–**k** The homogenates from contralateral (con) and ipsilateral (ipsi) cortical brains were subjected to Western blot analysis using indicated antibodies (**i**) and quantification analyses (**j**, **k**). Note that the membrane was blotted with the anti-Caspase 3 first, and then reblotted with anti-β-actin. **l** Mouse behavior tests to access the brain injury–associated deficits. The modified neurological severity score (mNSS) test, adhesive removal, and foot fault assays were described in the “Methods” section. Data presented are mean ± SEM; *n* = 10/group; *P < 0.05

To access microglial VPS35’s function in ischemia/stroke, we took advantage of the microglial VPS35 cKO mice and examined their response to photothrombotic cortical stroke injury. Specifically, microglial VPS35 cKO mice, named as VPS35^CX3CR1^ (Vps35^f/f^; CX3CR1-CreER), and their control (VPS35^f/f^ and/or CX3CR1-CreER) mice at age of postnatal (P) 15 were injected with tamoxifen for 4 times (once per day). Thirty days after the last tamoxifen injection, the mice were subjected to photothrombotic cortical stroke injury (~ P50) and examined 3 days post-injury (~ P53) (see Fig. 1a, b). VPS35 was depleted in primary microglia from adult VPS35^CX3CR1^ mice treated with tamoxifen, but not control mice, by both immunofluorescence staining and Western blot analyses (data not shown). As shown in Fig. 1c–f, photothrombotic ischemia induced a local cortical brain injury in both control and VPS35^CX3CR1^ mice. To our surprise, the infarct volume in cortical brains of VPS35^CX3CR1^ mice was much less than that of control mice (Fig. 1c–f), implicating a protective effect by microglial VPS35 deficiency.

To further test this view, we examined ischemia/stroke-induced neuronal death by co-immunostaining analysis of cleaved caspase-3 (a marker for apoptotic cells) and NeuN (a marker for neurons) in the infarct area. The cleaved caspase-3-positive cells in the ischemic zone/core were obviously detected in the infarct area of control mice, but they were lower in VPS35^CX3CR1^ mice (Fig. 1g, h). Western blot analysis showed increased cleaved caspase-3 levels in the ischemic zone of the control, but not mutant, cortical brains (Fig.1i–k). These results are in line with the view for a less cortical brain injury response in VPS35^CX3CR1^ mice.

As the infarct area was located near the sensorimotor cortex, we tested if the ischemic injury affects motor function, which is evaluated by use of a modified neurological severity score, adhesive removal test, and foot fault test. Motor functional impairments were indeed less server in VPS35^CX3CR1^ mice with photothrombotic cortical injury, compared with that of control mice (Fig. 1l). Together, these results suggest an attenuation of ischemia/stroke-induced cortical brain injury in microglial VPS35-deficient mice.

**Fig. 2:**
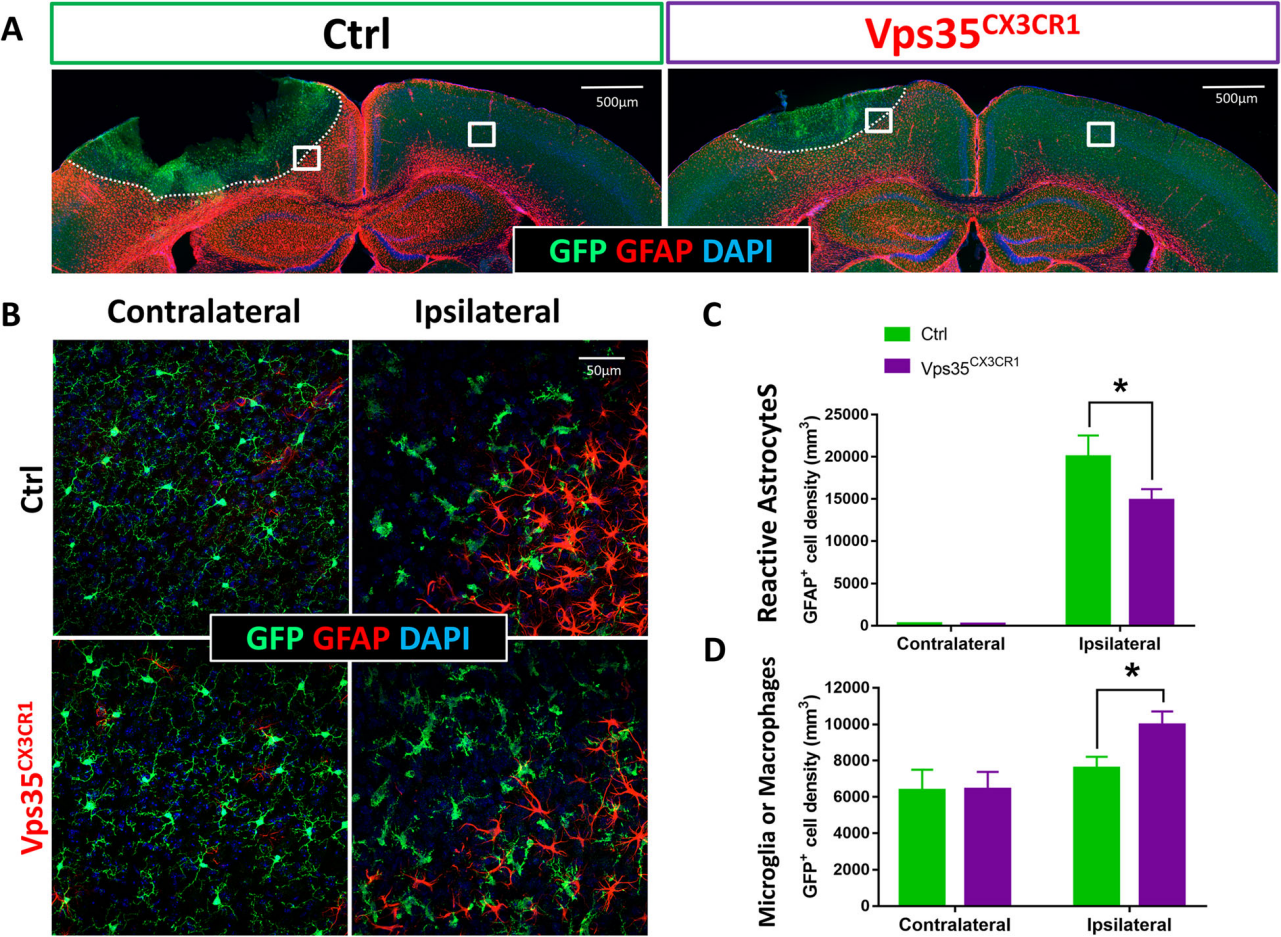
Decrease of reactive astrogliosis in VPS35^CX3CR1^ cortex after ischemic stroke. As described in the legend of Fig. 1a, the control (CX3CR1-CreER) and VPS35^CX3CR1^ mice were injected with tamoxifen at P15, induced photothrombotic stroke at P50, and sacrificed 3 days after the stroke. Their brain sections were subjected to co-immunostaining analyses using indicated antibodies. a, b Representative images. GFAP (glial fibrillary acidic protein), **a** marker for reactive astrocytes. **b** Amplified images from the white square marked regions in (**a**). **c**, **d** Quantification analyses (mean ± SEM; *n* = 4/group; *P < 0.05)

Neuronal death/injury often leads to an increase of reactive astrocytes, a key event for brain inflammation [27]. We then examined glial fibrillary acidic protein (GFAP) marked reactive astrocytes in control and VPS35^CX3CR1^ cortical brain in response to ischemia/stroke. GFAP^+^ astrocyte density was increased in the peri­infarct area of the control cortex 3 days post-injury (Fig. 2a). Compared with the control group, GFAP^+^ astrocyte density was much lower in the mutant peri­infarct area (Fig. 2b, c), suggesting a decrease in reactive astrogliosis in VPS35^CX3CR1^ cortex, providing additional support for an attenuated ischemic stroke injury in the mutant cortex.

### Increased microglial density without a reduction of CX3CR1-driven GFP expression in microglial VPS35-cKO mice in response to cortical ischemia/stroke

**Fig. 3:**
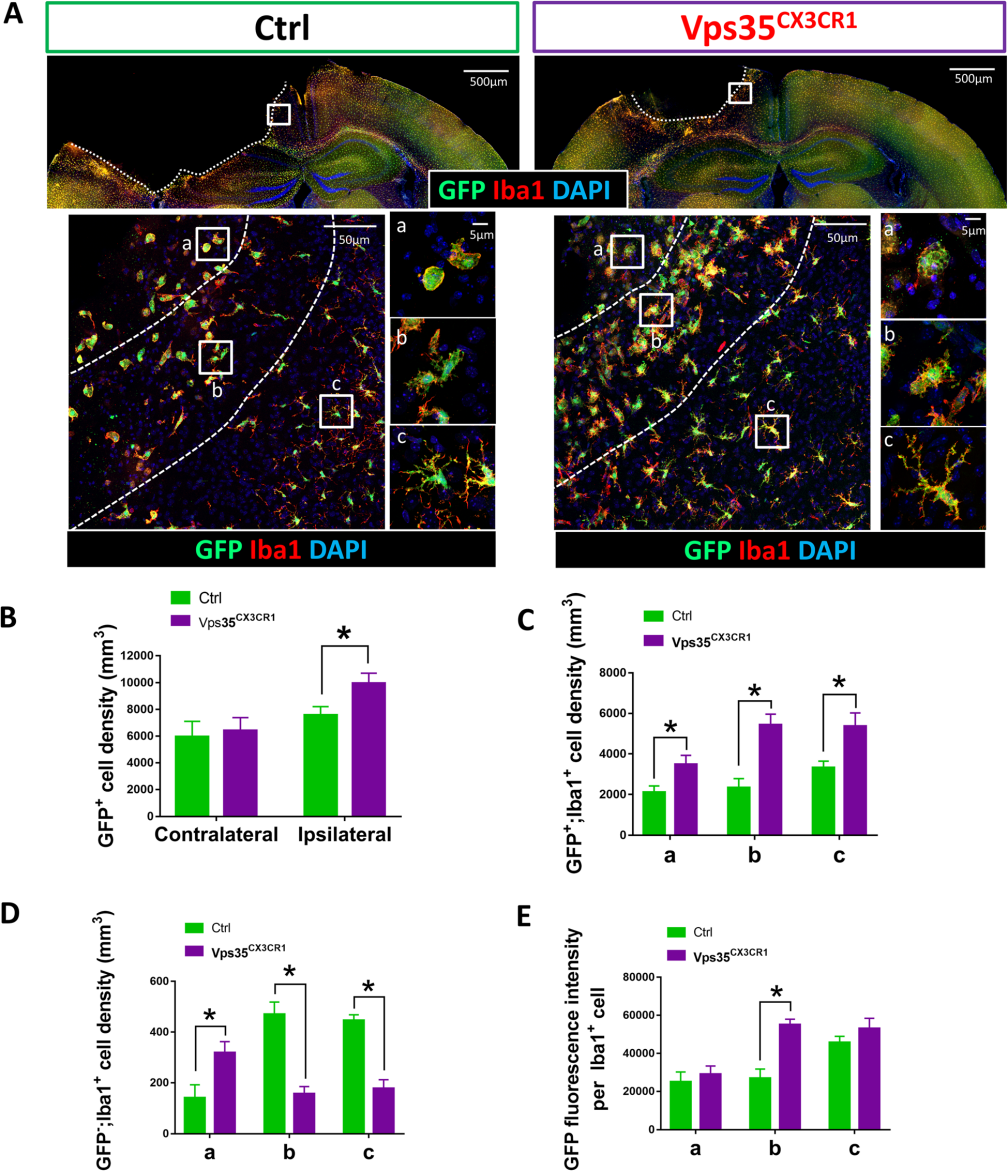
Altered microglial morphology and distribution in VPS35^CX3CR1^ cortex after ischemic stroke. The ischemic stroke experiment was carried out as described in the legend of Fig. 1a. Three days after the stroke, mouse brain sections were subjected to co-immunostaining analyses using indicated antibodies. **a**, **b** Representative images. Iba-1, a marker for microglia/macrophages. **c**, **d** Quantification analyses. Cell densities for GFP^+^;Iba1^+^ and GFP^−^(negative);Iba1^+^ microglia/macrophages were shown (mean ± SEM; *n* = 5/group; *P < 0.05). **e** GFP fluorescence intensity per Iba1^+^ cell was shown (mean ± SEM; *n* = 5/group; *P < 0.05)

To understand how microglial VPS35 deficiency results in a reduced cortical ischemia/stroke injury response, we examined microglial response to the stroke. In VPS35^CX3CR1^ or CX3CR1-CreER mice, there is enhanced YFP expression that is under the control of CX3CR1 promotor, providing a valued marker for microglial cells and macrophages [21]. We took advantage of this property and first examined GFP^+^ cell density in response to ischemia/stroke. In contrast from GFAP^+^ astrocytes, the total GFP^+^ cell density in ipsilateral peri­infarct area appeared to be higher in VPS35^CX3CR1^ mice than that of controls (CX3CR1-CreER mice treated with tamoxifen), whereas GFP^+^ cell density in contralateral side of cortex was comparable with that of controls (Fig. 2b–d). We then examined microglial properties in response to the injury by co-immunostaining analysis using antibodies against GFP and Iba1 (a marker for microglia/macrophages) (Fig. 3). In addition to the increase of GFP^+^ cell density, GFP^+^;Iba1^+^ cell density appeared to be higher at the ipsilateral peri­infarct area of the mutant cortex (Fig. 3a–c). However, careful analysis of GFP^−^(negative); Iba1^+^ cell densities in different regions (a, b, and c) of peri­infarct area showed a regional difference. In (a) region that close to the injury core, the GFP^−^;Iba1^+^ cell density in the mutant mice was significantly higher than that of control mice (Fig. 3a and d). In the (b) and (c) regions that are distally to the injury core, GFP^−^;Iba1^+^ cell densities in the mutant mice were lower than that of control mice (Fig. 3a and d), and the GFP^+^;Iba1^+^ double positive cell densities in both (a) and (b) regions were higher in the mutant peri­infarct area (Fig. 3c). These results suggest that VPS35 loss in microglia results in a cell autonomous increase of microglial/macrophage density (GFP^+^;Iba1^+^) and a cell non-autonomous decrease in GFP^−^; Iba1^+^ microglial/macrophage density in response to the injury, implicating regional difference in microglia/macrophage subtypes.

### Lower macrophage-like or DAM-like microglia in microglial VPS35-cKO mice in response to ischemic stroke

**Fig. 4:**
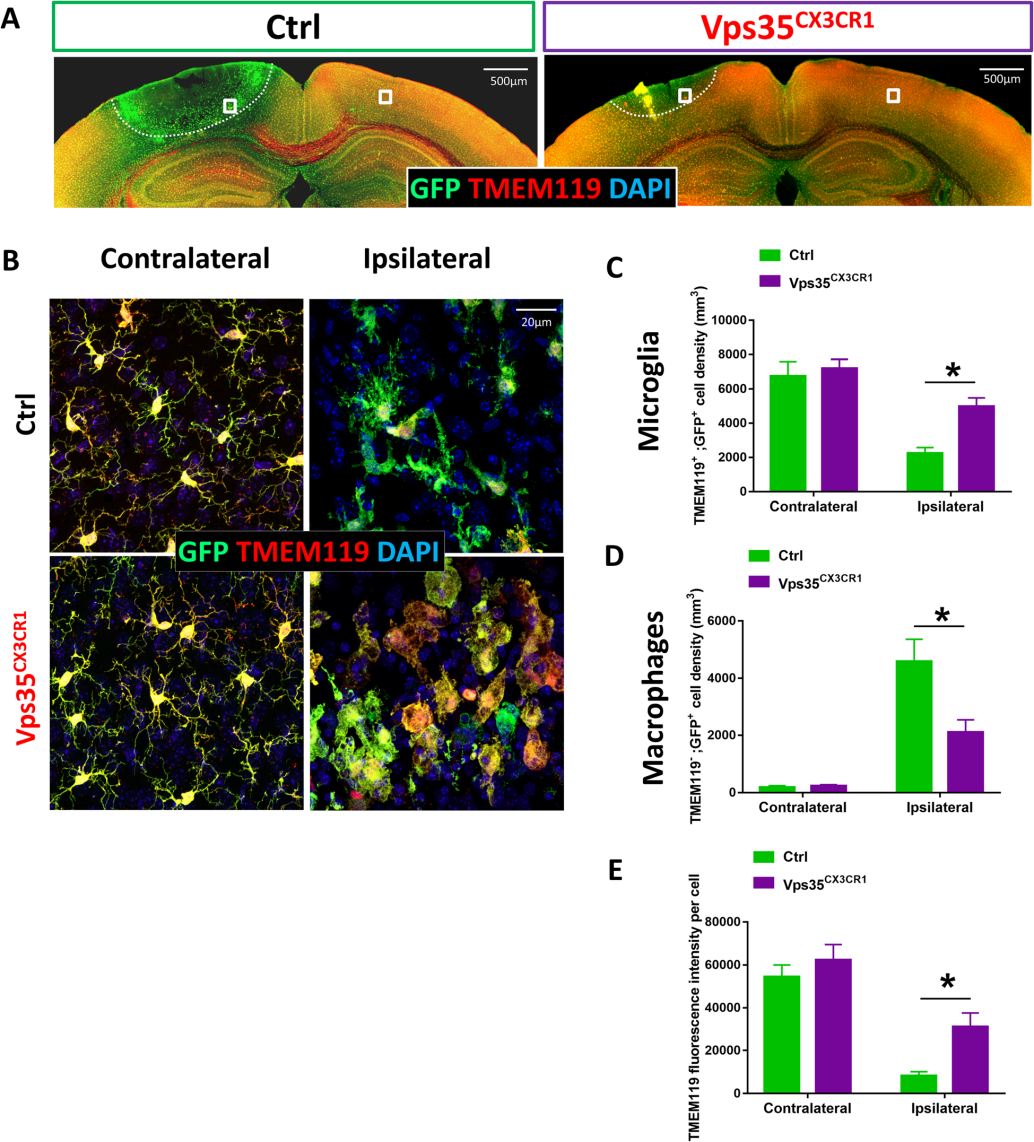
Altered microglia/macrophage ratio in VPS35^CX3CR1^ cortex after ischemic stroke. The ischemic stroke experiment was carried out as described in the legend of Fig. 1a. Three days after the stroke, mouse brain sections were subjected to co-immunostaining analyses using indicated antibodies. **a**, **b** Representative images: bar, 500 μm (**a**); bar, 20 μm (**b**). TMEM119, a marker for resting microglia. **c**–**e** Quantification analyses (mean ± SEM; *n* = 5/group; *P < 0.05)

It is of interest to notice recent reports for identification of DAM, a unique subtype of microglia, by single cell RNA-seq analysis [28]. DAM can be induced by multiple disease conditions, including stroke [28]. There are two stages of DAM: stage 1 is marked with altered gene expressions, such as reduced CX3CR1 and TMEM119, and increased Ctsd and ApoE (Apolipoprotein E); and stage 2 is featured with increased lipoprotein lipase (LPL), Trem2, and Cst7 [6]. The reduced CX3CR1-driven GFP in (b) region of the control, but not mutant, peri­infarct area (Fig. 3a) led us to ask whether stroke-induced stage 1 DAM might be impaired in VPS35^CX3CR1^ mice. We thus tested this view by immunostaining analysis of TMEM119, which is decreased in stage 1 DAM [6], but also is a marker for resting microglia [29]. Co-immunostaining analysis showed a marked reduction in GFP^+^;TMEM119^+^ double positive cell density in control peri­infarct area (Fig. 4b). However, such a reduction in GFP^+^;TMEM119^+^ cell density in response to the stroke was diminished in the mutant peri­infarct area as compared with that of controls (Fig. 4c). These results thus are in line with the view for an impairment in stage 1 DAM induction in VPS35^CX3CR1^ cortex by stroke. At the same time, these results also suggest that lesser “macrophage-like cells” (GFP^+^;TMEM119^−^ cells) were invaded into the mutant cortex after stroke.

**Fig. 5:**
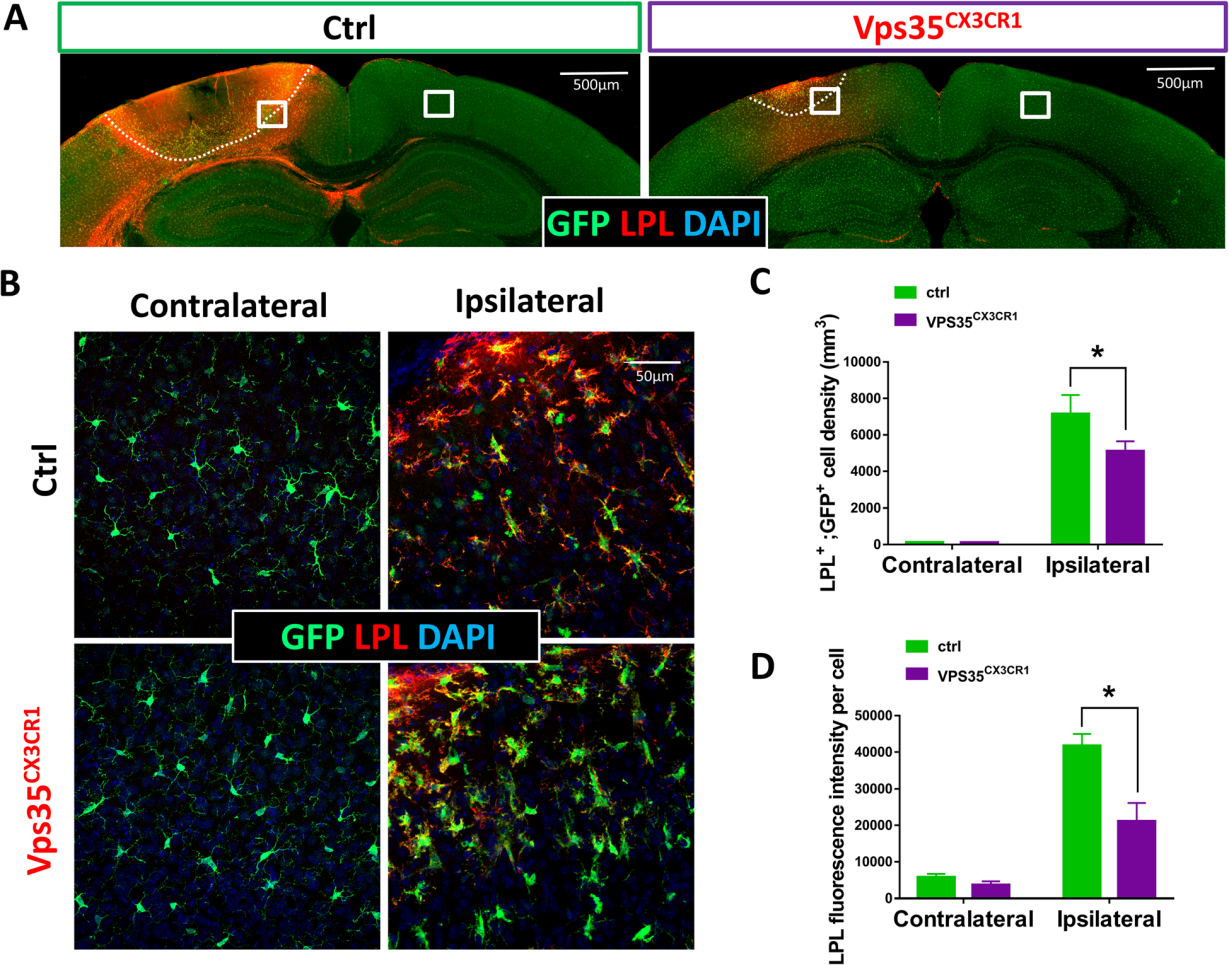
Decreased damage-associated microglia (DAM) in VPS35^CX3CR1^ cortex after ischemic stroke. The ischemic stroke experiment was carried out as described in the legend of Fig. 1a. Three days after the stroke, mouse brain sections were subjected to co-immunostaining analyses using indicated antibodies. **a**, **b** Representative images: bar, 500 μm (**a**); bar, 50 μm (**b**). LPL (Lipoprotein Lipase)-a marker for DAM (damage-associated microglia). **c**, **d** Quantification analyses (mean ± SEM; *n* = 5/group; *P < 0.05)

In addition to TMEM119, we examined LPL, a maker for stage 2 DAM [6]. LPL^+^;GFP^+^ cell density was also lower in VPS35^CX3CR1^ mice than that of control mice in response to the stroke injury (Fig. 5a, b). There was no change in the contralateral side of cortex between control and mutant mice (Fig. 5c, d). These results provide additional support for the view of a reduction in stroke-induced DAM in VPS35^CX3CR1^ cortex.

### Reduced ischemic stroke-induced pro-inflammatory, but increased anti-inflammatory, microglia in microglial VPS35-deficient cortex

**Fig. 6:**
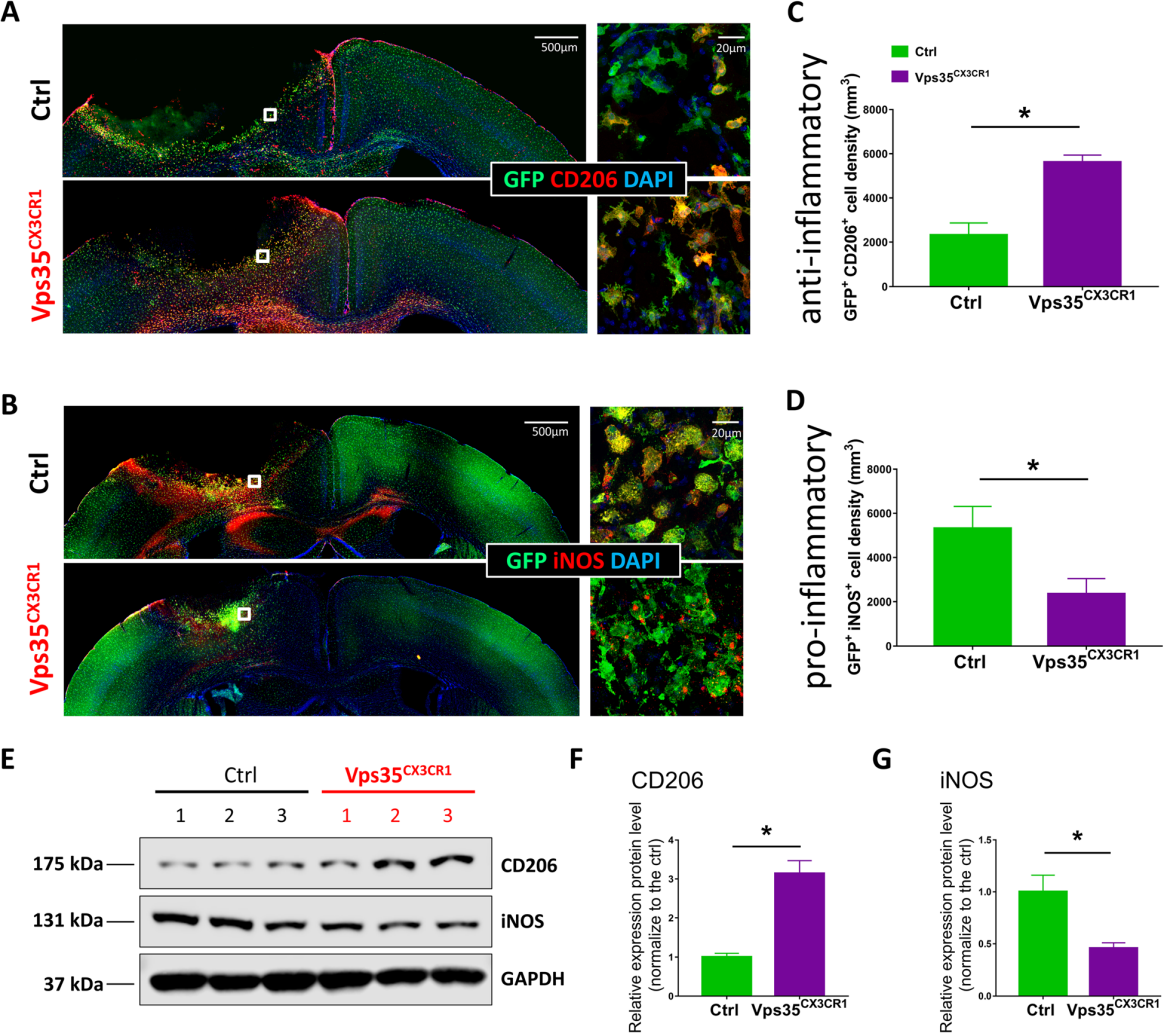
Increased anti-inflammatory, but decreased inflammatory, microglia in VPS35^CX3CR1^ cortex after ischemic stroke. The ischemic stroke experiment was carried out as described in the legend of Fig. 1a. Three days after the stroke, mouse brain sections were subjected to co-immunostaining analyses using indicated antibodies (**a**–**d**), and the homogenates from indicated area were subjected to Western blot analysis (**e**–**g**). **a**, **b** Representative images.CD206 (an anti-inflammatory microglia-specific marker); iNOS (a marker for pro-inflammatory microglia); GFP (a reporter of CX3CR1^+^ microglia/macrophage). **c**, **d** Quantification analyses (mean ± SEM; *n* = 5/group; *P < 0.05). **e**–**g** Western blot analysis of homogenates of cortex from control and mutant mice using indicated antibodies (mean ± SEM; *n* = 3/group; *P < 0.05)

Pro-inflammatory and anti-inflammatory microglia/macrophages are characterized by different gene expressions and have differential functions or contributions to the stroke [30]. We thus examined both pro-inflammatory and anti-inflammatory-like microglia/macrophages in control and VPS35^CX3CR1^ mice after ischemic injury by immunofluorescence staining analyses of marker proteins for both types of microglia/macrophages. The pro-inflammatory microglia/macrophages are marked by iNOS; and the anti-inflammatory microglia/macrophages can be labeled with CD206 [31]. Unexpectedly, the density in CD206^+^;GFP^+^ double positive cells (Fig. 6a and c) was significantly higher, but the density in iNOS^+^;GFP^+^ cells was significantly lower, in the mutant peri­infarct area, as compared with those of control mice (Fig. 6b and d). The increased CD206 and decreased iNOS protein levels in the mutant peri­infarct area were also detected by Western blot analysis (Fig. 6e–g). These results suggest a decrease of pro-inflammatory, but an increase of anti-inflammatory, microglia/macrophages in VPS35^CX3CR1^ mice in response to the injury.

**Fig. 7:**
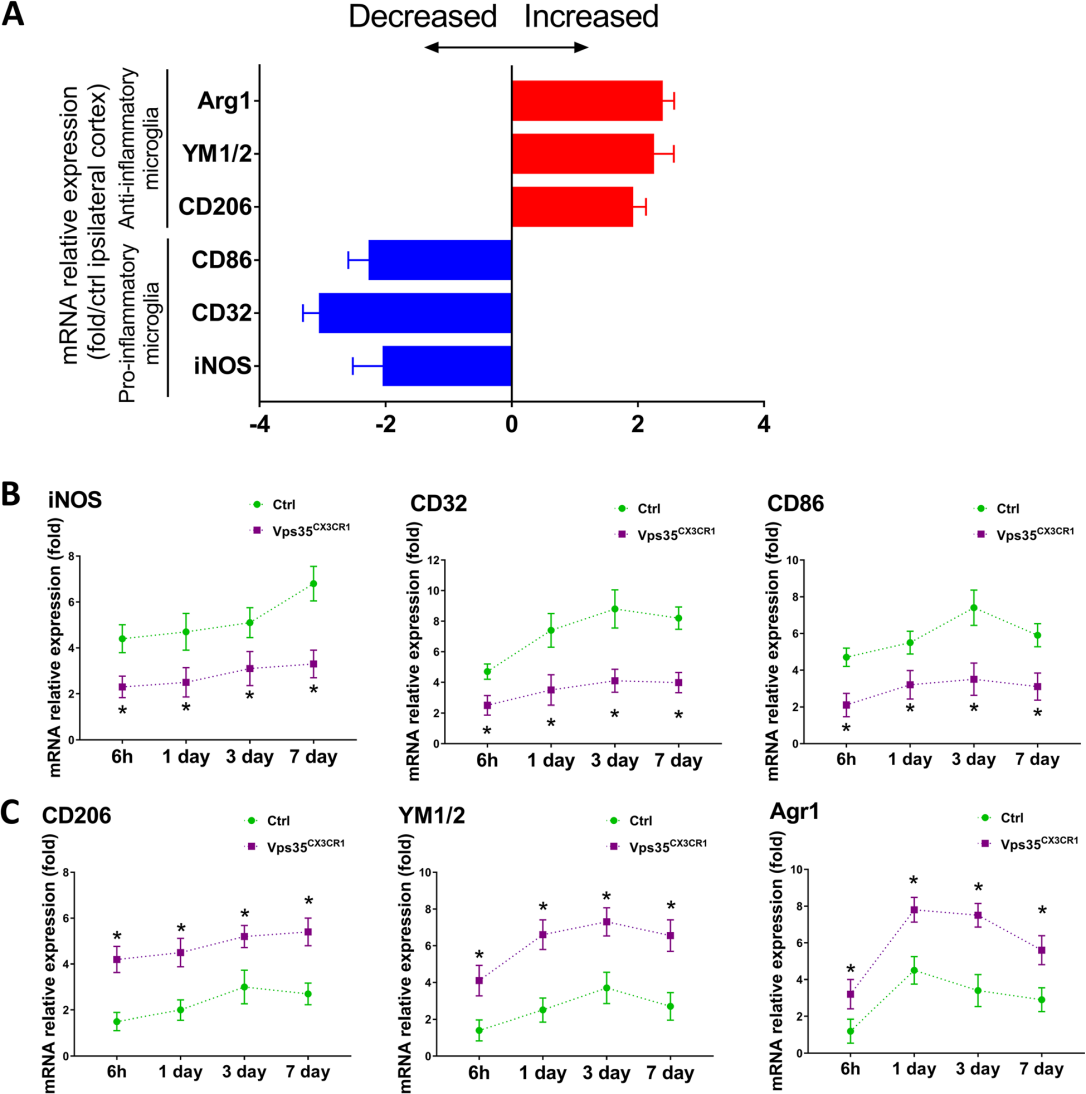
Expression of factors associated with inflammatory and anti-inflammatory microglia in VPS35^CX3CR1^ cortex after ischemic stroke. RT-PCR analysis was performed using total RNAs extracted from ischemic brains (of control and Vps35 mutant mice) at 6 h and 1, 3, and 7 days after stroke and primers for genes associated with anti-inflammatory microglia (e.g., Arg1, YM1/2, and CD206) or pro-inflammatory microglia (e.g., CD86, CD32, and iNOS). **a** Increased expression of factors associated with anti-inflammatory microglia, but decreased expression of factors associated with inflammatory microglia, in VPS35 mutant cortex 3 days after stroke. Data are expressed as fold change (mutant/controls). *n* = 4 per group. *P < 0.05. **b** Reduced expression of factors associated with inflammatory microglia in VPS35 mutant cortex at indicated time point post-stroke. **c** Increased expression of factors associated with anti-inflammatory microglia in VPS35 mutant cortex at indicated time point post-stroke. Data in b and c are expressed as fold change vs sham-operated controls. *n* = 4 per group. *P < 0.05

To further test this view, we examined additional pro- or anti-inflammatory microglia/macrophage-associated gene expression profiles in control and mutant mice in response to the stroke by RT-PCR analysis. The mRNA samples from peri­infarct area, at indicated time point post-stroke, were examined. Indeed, the mRNA levels of genes for pro-inflammatory microglia (e.g., iNOS, CD32, CD86) were all significantly lower in the mutant groups than those of controls (Fig. 7a, b). In contrast, the mRNA levels of genes for anti-inflammatory microglia, including CD206, YM1/2, Arg1, were all significantly higher in the mutant groups than those of controls (Fig. 7a and c). These results thus support the view that the loss of microglial VPS35 may facilitate the polarization from pro- to anti-inflammatory microglia/macrophage after ischemic stroke.

**Fig. 8:**
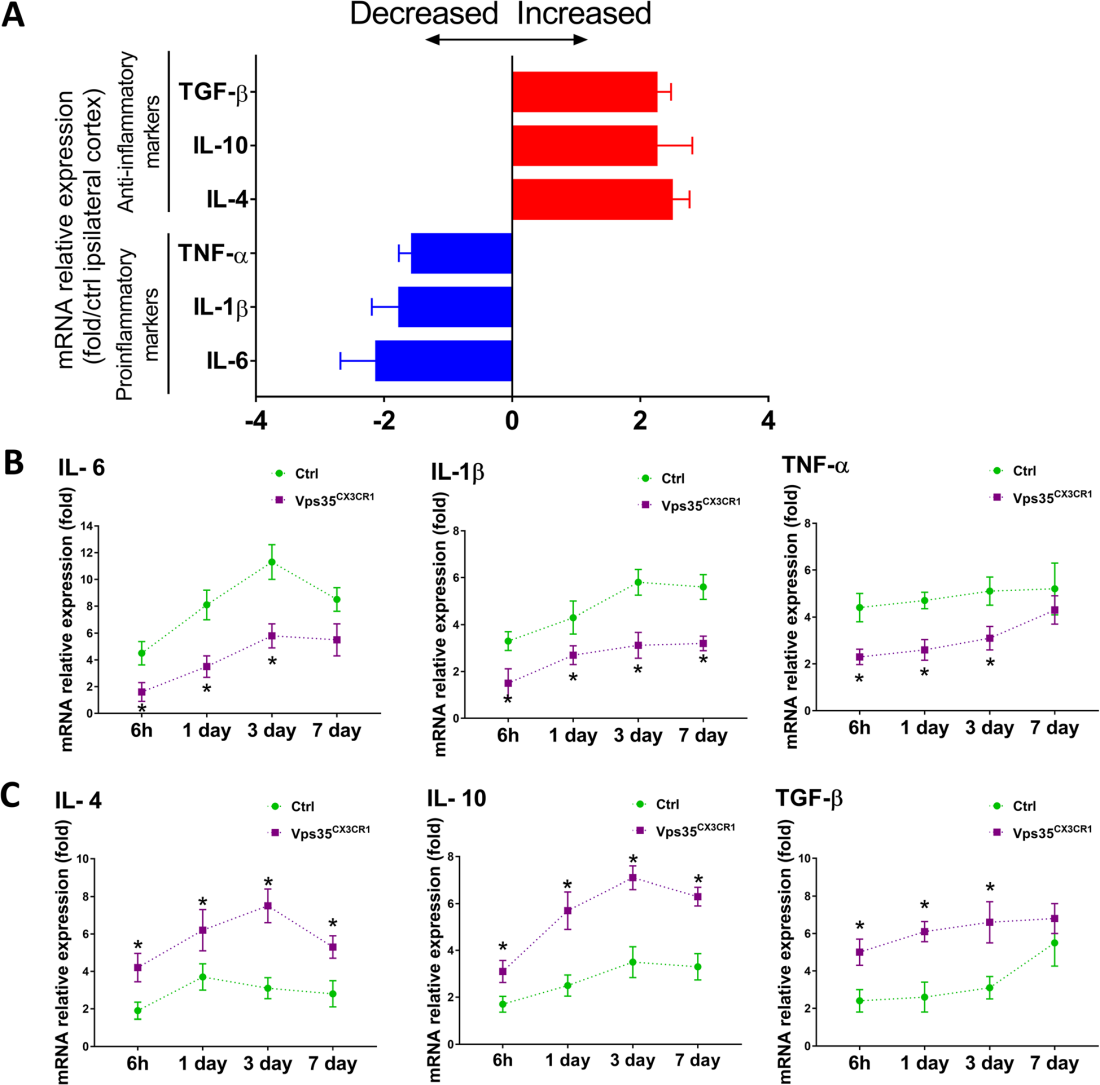
Expression of inflammatory cytokines in VPS35^CX3CR1^ cortex after ischemic stroke. RT-PCR analysis was performed using total RNA extracted from ischemic brains (of control and VPS35 mutant mice) at 6 h and 1, 3, and 7 days after stroke as described in the legend of Fig. 7. **a** Relative expression of mRNAs for indicated cytokines at day 3 after stroke. Data are expressed as fold change of mutant vs controls. *n* = 4 per group. *P < 0.05. **b** Expression of mRNAs for IL-6, IL-1β, and TNF-α pro-inflammatory cytokines. **c** Expression of mRNAs for IL-4, IL-10, and TGF-β anti-inflammatory cytokines. Data in b and c are expressed as fold change vs sham-operated controls. *n* = 4 per group. *P < 0.05

It is believed that the pro-inflammatory microglia/macrophages mediate neurotoxic response; and the anti-inflammatory microglia/macrophages are neuroprotective [7, 30, 32]. We thus further examined the expression of inflammatory cytokines by RT-PCR in control and VPS35^CX3CR1^ mice after ischemic injury. The levels of pro-inflammatory cytokines (e.g., IL-6, IL-1β, and TNF-α) were all induced by stroke in control mice (Fig. 8a, b). However, such induced pro-inflammatory cytokines were all diminished in the mutant mice (Fig. 8a, b). On the other hand, the mRNA levels of anti-inflammatory cytokines, such as IL-4, IL-10, and TGF-β, were lower in control mice than those of mutant mice (Fig. 8a and c). These results provide additional evidence that microglial VPS35 loss may reduce pro-inflammatory microglia but increase anti-inflammatory microglia after stroke.

### Decreased ischemia/stroke-induced CX3CR1 proteins levels in VPS35^CX3CR1^ cortical brain

**Fig. 9:**
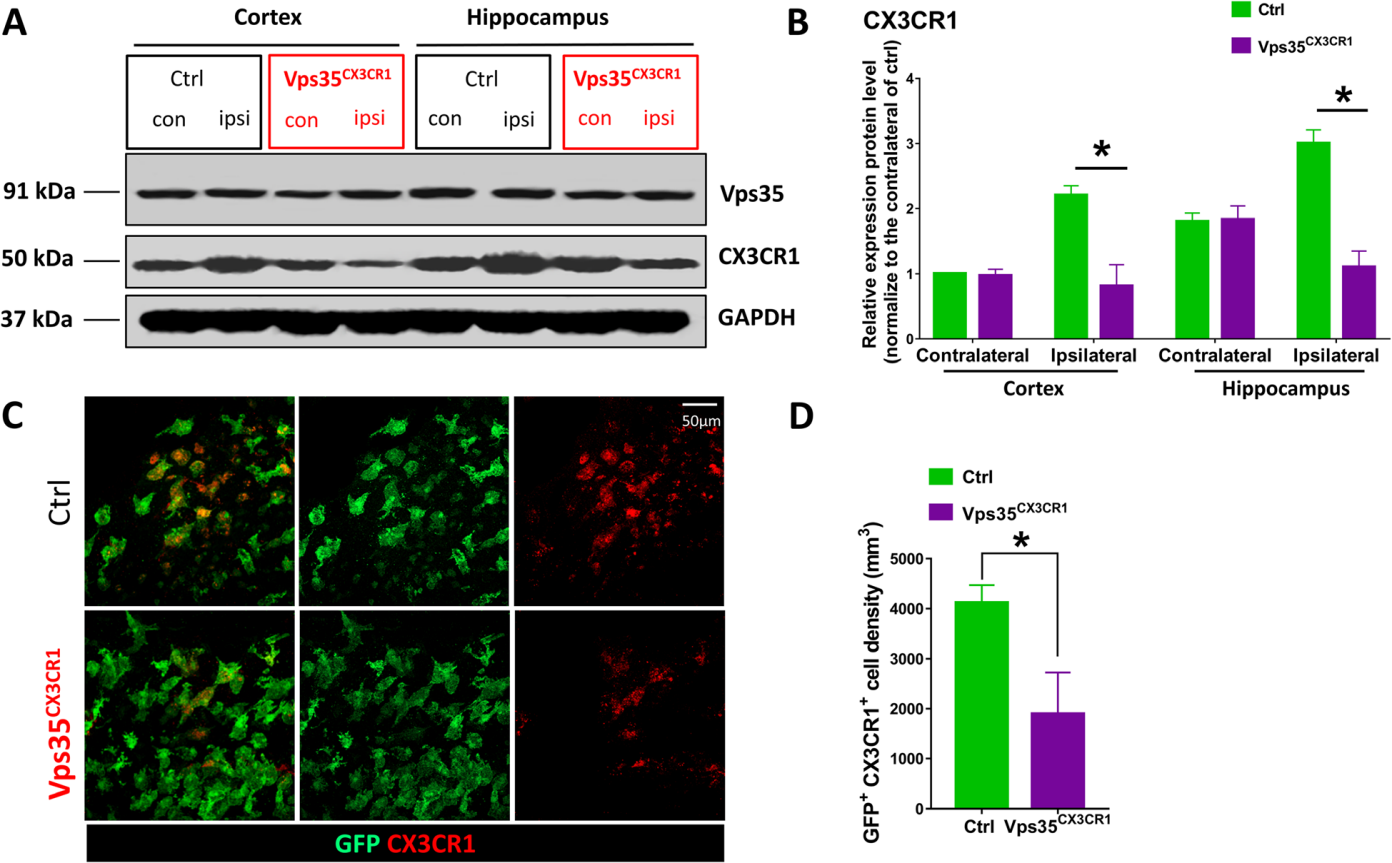
Decrease of CX3CR1 expression in VPS35^CX3CR1^ cortex after ischemic stroke. a Western blot analysis. At day 3 after stroke, the homogenates from contralateral (con) and ipsilateral (ipsi) cortical brains and hippocampus were subjected to Western blot analysis using indicated antibodies. **b** Quantification analysis of data in (**a**) for CX3CR1 protein levels (mean ± SEM; *n* = 3/group; *P < 0.05). c Co-immunostaining analysis using antibodies against CX3CR1 and GFP in ipsilateral cortical peri­infarct area. **d** Quantification analyses of data in (**c**) (mean ± SEM; *n* = 3/group; *P < 0.05)

It is of interest to note that the phenotypes in VPS35^CX3CR1^ mice in response to the injury resemble in certain degree to those in CX3CR1^−/−^ mice [33–35], which also show increased anti-inflammatory microglia/macrophages and neuroprotective effects in response to ischemic cortical brain injury. We thus wondered if CX3CR1 receptor level is altered in VPS35 mutant brain. Western blot analysis of cortical brain and hippocampus did not show obvious difference in CX3CR1 protein levels between control and VPS35 mutant mice. However, upon stroke injury, CX3CR1 levels were increased in injury side of cortex and hippocampus in control mice (Fig. 9a, b). Such stroke-induced CX3CR1 protein levels were abolished in VPS35^CX3CR1^ mice (Fig. 9a, b). These results suggest that CX3CR1 protein was downregulated in the mutant cortex in response to stroke. We further tested this view by co-immunostaining analysis using antibodies against CX3CR1 and GFP (Fig. 9c, d). In the injury side of cortex, CX3CR1^+^ GFP^+^ double positive microglia/macrophages were much more in the control cortex than that in the mutant cortex, in line with the results by Western blot.

## Discussion

VPS35 is known to play a key role in endosomal trafficking and cargo-selective function. Dysfunctional VPS35 increases a risk for neurodegenerative diseases, including Alzheimer’s disease (AD) and Parkinson’s disease (PD) [36]. Stroke is also believed to be an environmental risk factor for AD [37, 38]. While VPS35 deficiency contributes to the pathogenesis of neurodegenerative disorders [10–14], its function in stroke remains largely unclear. Here, we provide evidence for microglial VPS35’s function in ischemic cortical stroke. To our surprise, microglial VPS35 loss appears to be neuroprotective in this event. In microglial VPS35 KO mice, the injury response to photothrombotic stroke is reduced, which include decreased infarct area, attenuated neuronal death and reactive astrogliosis, and better sensorimotor regulated behavior functions. Further studies of microglial response to the stroke showed reduced pro-inflammatory but increased anti-inflammatory type of microglial polarization, in the mutant mice. Thus, the pro-inflammatory cytokine expression is downregulated, but the anti-inflammatory gene expression is upregulated in the mutant mice after the stroke. Finally, stroke-induced CX3CR1 protein levels are abolished in microglial VPS35-deficient mice. Taken together, these results suggest that microglial VPS35 is likely to be necessary for stroke-induced DAM and pro-inflammatory microglial activation, and both type of microglia might be “neurotoxic” in the condition of stroke induced injury.

Microglia play key roles in the physiology and pathology of the central nervous system, including ischemic brain injury. Microglial cells are not a uniform cell population, and they respond quickly with changes in transcriptional regulation and morphology to specific environmental factors, which include tissue injury (timing and degree), brain regions (e.g., cortex vs hippocampus), and age [39, 40]. Activated microglia/macrophages may have protective or harmful effects after ischemia injury, and these different functions may be due to different subtypes of microglia/macrophages, such as pro- or anti-inflammatory state [41]. Pro-inflammatory microglial activation is believed to be cytotoxic phenotype, which is characterized by the production of nitric oxide, reactive oxygen species, and pro-inflammatory cytokines. In contrary, anti-inflammatory microglia are associated with the promotion of debris removal, angiogenesis, and tissue repairing [37, 39, 40]. In line with this view are our observations of decreased iNOS^+^ microglia and reduced expression of pro-inflammatory cytokines in VPS35 mutant brains after ischemic stroke.

Recent single-cell RNA-seq analysis has identified DAM, a unique type of microglia that is induced in multiple disease conditions, including stroke [6, 29]. LPL is a marker for type 2 DAM [6]; however, it is also reported to be higher in anti-inflammatory microglia in response to EAE (experimental allergic encephalomyelitis) [42]. We found a decrease in LPL^+^ DAM, but an increase in CD206^+^ microglia (anti-inflammatory-like), in VPS35 mutant cortex after stroke (Figs. 5 and 6). Such a difference might be due to different experimental conditions (stroke vs EAE). In stroke condition, DAM may be in association with “neurotoxic” microglia (or pro-inflammatory), but under the condition of EAE, DAM, in association with beneficial microglia (or anti-inflammatory), mediate effect against EAE-induced inflammation. Further studies examining additional markers for both DAM and anti-inflammatory-microglia are necessary to test this view. Together, our results suggest associations of the reduced stroke injury in VPS35 mutant brain with decreases in iNOS^+^ microglia and pro-inflammatory cytokines’ expression, and an increase in CD206^+^ microglial activation. However, the molecular mechanisms underlying microglial VPS35 regulation of DAM/microglial polarization remain unclear, which need further investigation.

It is of interest to note previous reports that the loss of CX3CR1 receptors in mice has neuroprotective effects in response to ischemic brain injury [33, 43]. In MCAO stroke model, CX3CR1-deficient mice show reductions in infarct size and neuron death [33, 35]. In the experimental model of spinal cord injury, lack of CX3CR1 induces neurological function protection [34]. These phenotypes are remarkable similar to the phenotypes observed in microglial VPS35-cKO mice. In addition to the reduced infarct area in response to the ischemic injury, both VPS35 and CX3CR1 mutant mice show increased anti-inflammatory, but not pro-inflammatory, microglial activation by stroke [35] (Fig. 6) and reduced stroke-induced expressions of pro-inflammatory cytokines [33, 40, 41] (Fig. 8). These observations suggest that like CX3CR1-deficiency, VPS35 loss in microglia appears to attenuate neuronal damage and improve recovery of function by reducing the recruitment and/or the activation of DAM or pro-inflammatory microglia and macrophages in response to stroke injury [6, 29, 41, 43]. It is noteworthy that in contrast from stroke injury, CX3CR1 deficiency in AD animal models enhances Aβand Aβassociated brain pathology [37, 44]; and such enhanced Aβphenotypes are also observed in VPS35-deficient mice [13]. The similarity of phenotypes between VPS35 and CX3CR1 mutant mice leads us to wonder if CX3CR1 receptor is a cargo of VPS35/retromer in microglia. Examining CX3CR1 protein levels by Western blot analysis showed no difference between control and microglial VPS35-cKO brains, but, stroke-induced CX3CR1 protein levels were only detected in control, but not in VPS35-mutant, brains (Fig. 9a). The increase in CX3CR1 protein levels (Fig. 9a), but decrease in CX3CR1 promotor-driven GFP expression in subset of microglia (Fig. 3a), in control brains by stroke implicates that the elevation of CX3CR1 protein levels is likely due to an enrichment in populations of microglia/macrophages in the injury side of brains, as compared with that in contralateral side. The reduction in stroke-induced CX3CR1 protein levels in VPS35 cKO brain suggests a role of microglial VPS35 in stabilizing CX3CR1 proteins in response to stroke. However, this view requires further investigations.

## Conclusions

In summary, we have found a protective effect on ischemia/stroke-induced cortical brain injury by microglial VPS35 deficiency. Such a protective effect may result from the decreased pro-inflammatory and anti-inflammatory microglial activation by stroke. Further investigations are necessary to test the view if CX3CR1 acts as a cargo of VPS35 in microglial cells to participate in stroke-induced injury response.

## Data Availability

All data generated or analyzed during this study are included in this published article.

## Abbreviations

VPS35: Vacuolar sorting protein 35
CX3CR1: CX3C chemokine receptor 1
DAM: Damage-associated microglia
TTC: 2,3,5-Triphenyltetrazolium chloride
RT-PCR: Real-time polymerase chain reaction
cKO: Conditionally knocking out
GFAP: Glial fibrillary acidic protein
LPL: Lipoprotein lipase
Aβ: Amyloid β
EAE: Experimental allergic encephalomyelitis
